# Declaring a tuberculosis outbreak over with genomic epidemiology

**DOI:** 10.1099/mgen.0.000060

**Published:** 2016-05-31

**Authors:** Hollie-Ann Hatherell, Xavier Didelot, Sue L. Pollock, Patrick Tang, Anamaria Crisan, James C. Johnston, Caroline Colijn, Jennifer L. Gardy

**Affiliations:** ^1^​ CoMPLEX, University College London Research Department of Infection and Population Health, University College London, London, UK; ^2^​ Department of Infectious Disease Epidemiology, Imperial College London, London, UK; ^3^​ Interior Health Authority, British Columbia, Canada; ^4^​ British Columbia Centre for Disease Control Public Health Laboratory Department of Pathology, and Sidra Medical and Research Center, British Columbia, Canada; ^5^​ Communicable Disease Prevention and Control Services, British Columbia Centre for Disease Control, and School of Population and Public Health, University of British Columbia, Vancouver, BC, Canada; ^6^​ Clinical Prevention Services, British Columbia Centre for Disease Control, Vancouver, BC, Canada; ^7^​ Department of Mathematics, Imperial College London, London, UK

**Keywords:** Tuberculosis, transmission, genomic epidemiology, phylogenetics

## Abstract

We report an updated method for inferring the time at which an infectious disease was transmitted between persons from a time-labelled pathogen genome phylogeny. We applied the method to 48 *Mycobacterium tuberculosis* genomes as part of a real-time public health outbreak investigation, demonstrating that although active tuberculosis (TB) cases were diagnosed through 2013, no transmission events took place beyond mid-2012. Subsequent cases were the result of progression from latent TB infection to active disease, and not recent transmission. This evolutionary genomic approach was used to declare the outbreak over in January 2015.

## Data Summary

1. Short read data for the 48 sequenced M. tuberculosis genomes has been deposited in the European Nucleotide Archive; accession number: PRJEB12764 (url - http://www.ebi.ac.uk/ena/data/view/PRJEB12764)
2. The commands used in reference alignment and variant calling are available as a text file from Figshare; DOI: 10.6084/m9.figshare.3153280 (url - https://figshare.com/articles/Declaring_a_tuberculosis_outbreak_over_with_genomic_epid emiology/3153280)
3. The final dataset of variants is available as a fasta file from Figshare; DOI: 10.6084/m9.figshare.3153280 (url - https://figshare.com/articles/Declaring_a_tuberculosis_outbreak_over_with_genomic_epidemiology/3153280)
4. The TransPhylo code, including the update written specifically for this study, is available from GitHub (url - https://github.com/xavierdidelot/TransPhylo)

## Impact Statement

We have previously described a method for inferring person-to-person transmission events from pathogen genome data; here, we describe an improvement to the method’s underlying epidemic model that allows us to infer the likely time at which a person was infected. Significantly, we describe how this updated approach was used in the real-time investigation of a large tuberculosis (TB) outbreak and how our results were used to declare an end to the outbreak. This is the first report of genomic data being used to declare a complex community outbreak over, suggesting a new role for the use of genomic data in TB control.

## Introduction

Genomics is revolutionizing public health practice (Kwong *et al.,* 2015). Mutational and evolutionary events within a pathogen population not only have consequences for the disease, but also present opportunities for understanding transmission and developing targeted public health interventions. Inferring person-to-person transmission from genomic data is one such example – genome sequencing has now helped identify individual infection events in multiple outbreaks at levels from hospital wards to communities to countries (Croucher & Didelot, 2015).

**Fig. 1.**
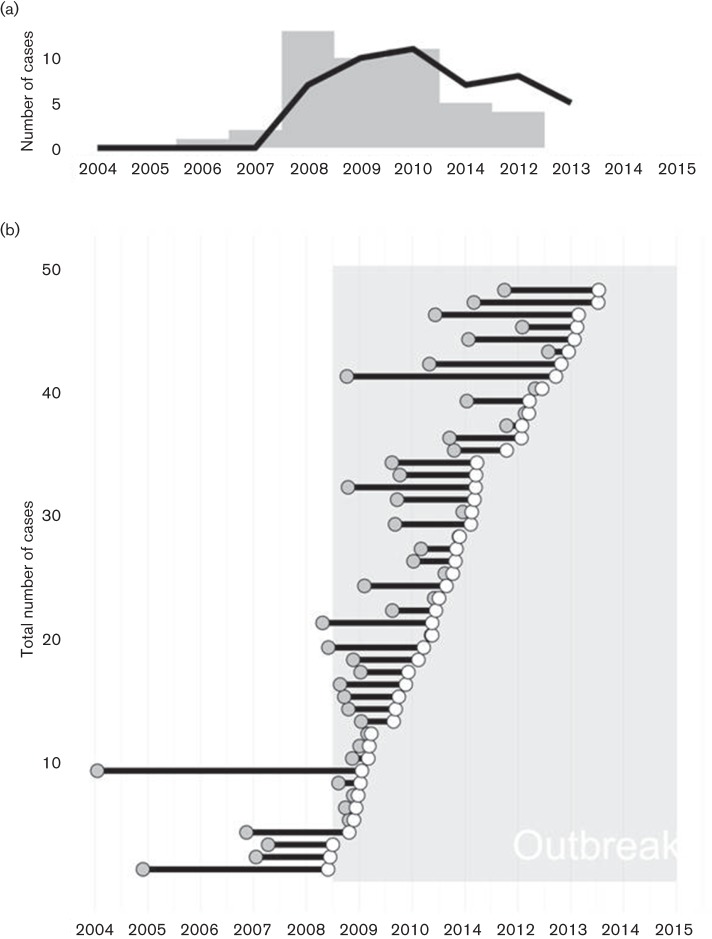
Timing of infections inferred from genomic data. (a) Epidemic curve for the outbreak based on time of diagnosis (black line) or *T_inf_*, the time of infection estimated by TransPhylo (grey bars). (b) Infected period for each case shown as a line originating at *T_inf _*(grey dot) and continuing until the case was diagnosed (white dot). The outbreak period (May 2008 – January 2015) is indicated with shading.

Transmission inference from genomic data uses mutations – fixed or minor variants (Worby *et al.,* 2014; Poon *et al*., 2016 ) – shared across outbreak isolates to identify putative infection events. We previously developed TransPhylo (Didelot *et al.,* 2014), a Bayesian method for inferring transmissions and their timing given mutational events captured in a time-labelled phylogeny, and used it to reconstruct transmissions between the first 33 cases (2008–2011) of a large tuberculosis (TB) outbreak. The outbreak in question began with the May 2008 diagnosis of a highly infectious client in a homeless shelter in British Columbia, Canada and peaked in 2010. Intensive case-finding in the community ultimately screened 2310 individuals and a total of 52 TB cases were diagnosed through December 2013 (Fig. 1a) (Cheng *et al.,* 2015).

In the absence of a formal definition, a TB outbreak is generally deemed over when transmission of the outbreak strain has stopped for >2 years; however, latent TB infection (LTBI) complicates declaring the end of an outbreak. Amongst individuals identified with LTBI, only 5–10% will progress to active disease, with most developing disease within two years of infection (World Health Organization, 2015); however, delayed progression occurring more than two years after infection is not uncommon. As incident case numbers begin to decline, TB controllers must differentiate LTBI cases acquired >2 years ago and only now progressing to active disease from new cases that were recently acquired, suggesting ongoing transmission.

One of TransPhylo’s outputs is *T_inf _*, the estimated time at which an individual was infected. *T_inf_* can differentiate delayed progression from new infection; however, TransPhylo’s underlying SIR model, and indeed all compartmental epidemic models, does not capture the true size of the susceptible population or the true variation in the infectious periods, which may affect inferred *T_inf _*values.

In September 2014, at the request of the Medical Health Officer leading the outbreak response, we analysed *Mycobacterium tuberculosis* genomes from 48 of the 52 cases to determine whether a decline in newly diagnosed cases truly signaled the end of the outbreak. We replaced TransPhylo’s SIR model with a branching model to better infer the timing of transmission amongst the 48 cases and asked whether cases diagnosed in 2013 were the result of recent transmission or delayed progression of an infection acquired earlier in the outbreak.

## Methods

As part of an earlier investigation during the outbreak, we sequenced 33 *M. tuberculosis* genomes from outbreak cases diagnosed between May 2008 and April 2011 (Didelot *et al.,* 2014). In September 2014, we sequenced genomes from a further 15 cases on the MiSeq platform, for a total of 48 genomes (GenBank accession no. PRJEB12764, Data Citation 1). Four of the 52 outbreak cases did not have an *M. tuberculosis *isolate available for sequencing as they were diagnosed out-of-province or on clinical grounds during a post-mortem.

Reads were mapped against the *M. tuberculosis *CDC1551 reference genome (GenBank accession no. NC_002755.2) using BWAmem (Li & Durbin, 2009) and variants called using samtools mpileup (Li *et al*., 2009); the commands we used are available in a text file from FigShare (DOI: 10.6084/m9.figshare.2077390, Data Citation 2). From the resulting VCF files, we removed all variant positions that were identical across all 48 genomes, leaving only variants that differentiate outbreak isolates. We filtered a matrix of these positions to remove variant positions within 150 bp of another variant position (suggesting misalignment to a low-complexity region), as well as variant positions without an mpileup QUAL score equal to 222 in at least one isolate. This left 28 positions across the 48 isolates, which were manually reviewed before further analysis; a FASTA file of these variants labeled with isolate sampling date (in the format ‘days since X’) is available at FigShare (DOI: 10.6084/m9.figshare.2077405, Data Citation 3). Variants were concatenated and analysed with BEAST (Drummond & Rambaut, 2007) and the resulting timed phylogeny was passed to TransPhylo for transmission timing inference.

We used a modified version of TransPhylo (https://github.com/xavierdidelot/TransPhylo, Data Citation 4) in which we replaced the existing SIR epidemic model with a branching model. The mathematics describing the branching model are detailed in a methods supplement (available in the online Supplementary Material).

## Results

Examining *T_inf_* for each of the 48 sequenced cases revealed that the last person-to-person transmission occurred in late July or early August 2012 (Fig. 1a, b). The eight cases diagnosed afterwards were all instances of delayed progression, with most of the transmission events leading to these cases occurring in 2010–2011. Indeed, the epidemic curves based on *T_inf_* and on diagnosis date (Fig. 1a) echo each other, with transmission concentrated largely before 2011 and diagnoses extending two years beyond that, underscoring the importance of early active case-finding and preventive therapy in a TB outbreak.

The average time from infection to diagnosis was 1.2 years (95 % CI ± 0.31; Fig. 1b), increasing from 0.98 (95 % CI ± 0.82) in 2009 to 1.97 (95 % CI ± 0.69) in 2013 despite continued intensive surveillance. This supports the hypothesis that later cases were largely due to delayed progression of infection acquired earlier in the outbreak and highlights the need for extensive follow-up of infected contacts and provision of LTBI preventive therapy.

**Fig. 2.**
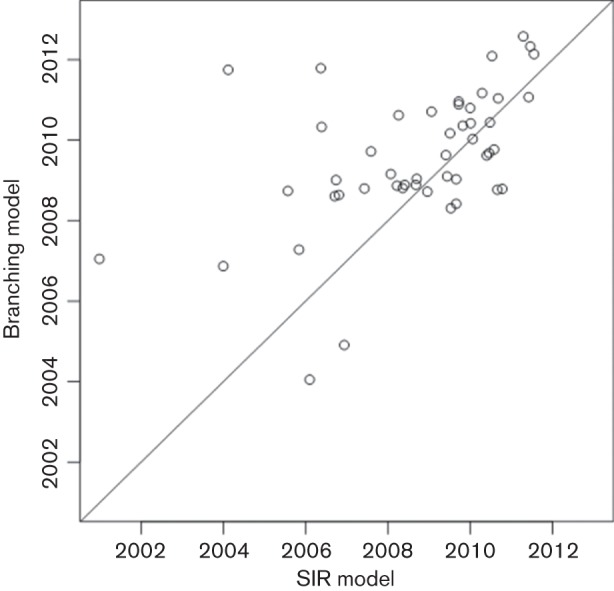
*T_inf_* estimated by a branching model versus SIR model.

We also compared the *T_inf_* values estimated here to those estimated using the original TransPhylo release (Fig. 2). Both the original SIR model and the updated branching model reported here support our observation of at least two years without a transmission event. While the two methods are somewhat similar in their *T_inf_* estimates for isolates transmitted later in the outbreak, there is substantial variation in early *T_inf_* values, with the original SIR model reporting transmissions as early as 2000. The values reported by the branching model are more consistent with the epidemiology of TB in the outbreak community, demonstrating the importance of incorporating an epidemic model that better reflects the biology underlying the epidemic process.

## Conclusion

We presented our findings to the Medical Health Officer and the Outbreak Management Team on 9 January 2015. After considering our genomic evidence indicating that no transmission of the outbreak strain had been detected since 2012 – thereby fulfilling the criteria for at least two years without a transmission event – and corroborating evidence from the ongoing epidemiological investigation, the outbreak was declared over on 29 January 2015 (Interior Health Authority, 2015).

This is the first demonstration that evolutionary genomic analysis can be used to declare a complex community outbreak over, suggesting a new role for public health genomics in not just identifying transmission events, but also in timing these events to better understand the dynamics of an outbreak and guide the real-time public health response.

## Supplementary Data

146 Supplementary File 1
