# Supplementary material for: Declaring a tuberculosis outbreak over with genomic epidemiology
Source: Microb Genom. 2016 May 31;2(5):e000060. doi: 10.1099/mgen.0.000060 (PMC5320671; doi:10.1099/mgen.0.000060)
Supplement: Supplementary file 1 [file mgen-02-60-s001.pdf]

Supplementary Methods

February 9, 2016

1 Branching Model

Our approach follows Didelot et al. (2014), using the idea of assigning each host to a connected subset of a fixed, timed, phylogenetic tree (a genealogy). This assignment can be considered as a "colouring" of the tree. Our method is an MCMC approach beginning from an initial valid colouring as in Didelot et al, updating the colouring, computing its likelihood, and using a Metropolis-Hastings accept/reject step. The colouring specifies who infected whom and when and the timed phylogenetic tree contains the times of sampling of the hosts. Accordingly, we now need to specify how the likelihood of a "colouring" is computed.

The key difference between our approach and that in Didelot et al. is that where Didelot et al. used a susceptible-infectious-recovered (SIR) model for the probability of the transmission process, we use a branching model. In this model, infected individuals cause a Poisson-distributed number of secondary infections (with mean connected to the generation time and the individual's infectious period). The time between becoming infected and infecting others is distributed according to a generation time distribution f_g. The time between becoming infected and being sampled is distributed according to a prior distribution f_s. As in Didelot et al, we assume that all infectious cases are known. We write the probability of the transmission tree T, conditional on the phylogenetic tree G, the in-host effective population size N_eg and the branching model parameters ϵ as

$$\mathbf{P}(T, \epsilon, N_eg|G) \propto \mathbf{P}(G|T, \epsilon, N_eg)\mathbf{P}(\epsilon, N_eg, T) \tag{1}$$

which we write as

$$\mathbf{P}(T, \epsilon, N_eg|G) \propto \mathbf{P}(G|T, N_eg)\mathbf{P}(T|\epsilon)\pi(\epsilon, N_eg), \tag{2}$$

where π represents the priors for ϵ and N_eg. The likelihood of the genealogical component G, $\mathbf{P}(G|T, N_eg)$, is as given in Didelot et al and is the product of the likelihoods of the (independent) genealogies inside each host under a fixed-size coalescent model.

The term $\mathbf{P}(T|\epsilon$ represents the probability of the transmission tree under the parameters of the branching model. We write this using the probability of the number of secondary infections k of each case (Poisson), the probability that the case was sampled at the specified time after infection (using f_s), and the probabilities of the infection times of the secondary infections using f_g. Since we assume that each case's infectious period ends at their time of sampling, we condition on the infection times of observed cases being prior to that time. This gives

$$\mathbf{P}(T|\epsilon) = \prod_{i=1}^{n} f_o(k_i) f_s(t^i_{samp} - t^i_{inf}) \prod_{j=1}^{k_i} \frac{f_g(t^j_{inf} - t^i_{inf})}{F_g(t^i_{samp} - t^i_{inf})} \tag{3}$$

where f_s, f_o and f_g represent the probability density functions for the sampling, offspring and generation time distributions and F_g is the cumulative distribution function for the generation time. Equation (3) describes the probability for each individual $i = 1, ..., n$ in the tree of having k_i offspring given how long they were infectious and the probability of infecting offspring j at t^j_{inf} and being sampled at t^i_{samp} both conditional on when they were infected, t^i_{inf}.

The expected number of infections depends on an individual's duration of infectiousness and the infectivity, which is related to the generation time (Equation (4)). We used a gamma-distributed generation time (f_g is gamma with parameters k_g and θ_g) so as to have a distribution more centered around the mean than an exponential, and a long tail that allows for individuals to reactivate older TB infections. We have the expected number of secondary infections of host i:

$$\mathbf{E}_i = \int_0^{t^i_{samp}-t^i_{inf}} R_0 f_g(\tau)d\tau \tag{4}$$

$$= \frac{R_0}{\Gamma(k_g)}\gamma\left(k_g, \frac{t^i_{samp}-t^i_{inf}}{\theta_g}\right) \tag{5}$$

We assume that the secondary infections from each individual occur as a Poisson process, and use Equation (4) as the mean of the offspring distribution, giving us a time-inhomogeneous Poisson process with intensity function $R_0 f_g(\tau)$ up to $\tau = t_{samp} - t_{inf}$ and 0 thereafter. We assume the sampling time also follows a gamma distribution (f_s is gamma with parameters k_s and θ_s) to allow for a variable latency period with individuals sampled (and their TB sequenced and included in the study) only after they have active TB disease. In total, the parameters for the are branching model are $\epsilon = \{R_0, k_g, \theta_g, k_s, \theta_s\}$ and these are held fixed at values $\{1.5, 2, 1, 1, 2\}$ (though in principle they could also be assigned prior distributions). The full description of the terms is:

$$\frac{f_g(t^j_{samp}-t^i_{inf})}{F_g(t^i_{samp}-t^i_{inf})} = \frac{\frac{1}{\theta_g^{k_g}}(t^j_{samp}-t^i_{inf})^{k_g-1}e^{-\frac{t^j_{samp}-t^i_{inf}}{\theta_g}}}{\gamma\left(k_g, \frac{t^i_{samp}-t^i_{inf}}{\theta_g}\right)} \tag{6}$$

$$f_o(k_i) = \frac{1}{k_i!}(\mathbf{E}_i)^{k_i}e^{-\mathbf{E}_i} \tag{7}$$

$$f_s(t^i_{samp}-t^i_{inf}) = \frac{1}{\Gamma(k_s)\theta_s^{k_s}}(t^i_{samp}-t^i_{inf})^{k_s-1}e^{-\frac{t^i_{samp}-t^i_{inf}}{\theta_s}} \tag{8}$$

2 References

1. Didelot X, Gardy J, Colijn C. 2014. Bayesian inference of infectious disease transmission from whole-genome sequence data. Mol Biol Evol. 31:1869-1879.
